# Digital Approaches to Early Screening for Dementia

**DOI:** 10.1002/alz70858_099507

**Published:** 2025-12-25

**Authors:** Adrian M Owen

**Affiliations:** ^1^ Western University, London, ON, Canada

## Abstract

The rise of digital tools ‐ online cognitive tests, AI‐powered diagnostics, smart watches, and other app‐based symptom trackers ‐ offers unprecedented opportunities for the early detection and improved management of conditions like Alzheimer's disease and other dementias. Digital platforms offer a number of advantages over more traditional paper‐and‐pencil approaches, including increased sensitivity, increased specificity, ease of administration, broader performance measures (e.g. response times, attempt counts, and error types), as well as the number and depth of cognitive domains assessed. We have recently developed a five‐minute, web‐based cognitive ‘screener’ for detecting the early signs of dementia. A machine learning approach was used to identify the two most informative cognitive tasks from an initial library of 12 possibilities, covering working memory, attention, reasoning and problem‐solving. The evaluation model used 22 features from these tasks, including reaction times and error rates, to predict if an individual was cognitively healthy or potentially impaired. The model was trained on data from over 8,000 healthy individuals and more than 3,000 patients aged 50+. It was further validated with a different group of 800 healthy individuals and 1,000 patients, achieving an accuracy rate of over 80%. In individuals clinically diagnosed with Alzheimer's disease, 100% were accurately flagged for further testing. The results confirm that digital cognitive screeners for dementia offer comparable or superior accuracy relative to their traditional paper‐and‐pencil counterparts, can be easily scaled for mass deployment, and are a cost‐effective and convenient approach to early detection.

